# Chronic Undiagnosed Brucellosis Presenting as Sciatica

**DOI:** 10.7759/cureus.13114

**Published:** 2021-02-03

**Authors:** Meletis Rozis, John Vlamis, Spyros G Pneumaticos

**Affiliations:** 1 3rd Orthopaedic Department, University of Athens, KAT Hospital, Athens, GRC

**Keywords:** brucellosis, osteomyelitis, sinus, abscess

## Abstract

Brucellosis is a common zoonotic disease in southern Europe. Although having the potential to harm several anatomic regions and systems, musculoskeletal manifestations are rare, usually involving the spine and the sacroiliac joints. In the literature, the reports of hip manifestations are sporadic. We present a case report of chronic, undiagnosed brucellosis indirectly affecting the hip joint.

A 51-years-old male patient was admitted to our department with acute onset sciatica. His medical history was remarkable for incomplete cauda equina syndrome of unknown etiology and concomitant dura mater disruption, creating local sinuses resulting at the right buttock. On radiological evaluation, we demonstrated multiple abscesses of the lower lumbar spine and the ipsilateral sacroiliac joint, along with sinuses communicating with the right hip joint capsule. Soft and osseous tissue cultures obtained from the area of the lesion were negative for common bacteria. Considering the patient's history, chronicity of the disease, and the lesional pattern, we suspected brucellosis as a possible etiological factor. Laboratory evaluation with the serum agglutination test confirmed the diagnosis. The patient denied the surgical treatment, so we proceeded with chronic suppression antibiotics schemes. On 12-month follow-up, the patient has no clinical signs of infection relapse; he has reasonable pain control and a normal gait.

Indirect hip infection due to chronic brucellosis is rare, and physicians should be very suspicious of the disease's characteristic radiological manifestations to reach a correct diagnosis.

## Introduction

Brucellosis is the most common zoonotic disease worldwide [1] caused by four species, *Brucella melitensis*, *Brucella abortus*, *Brucella canis*, and *Brucella suis *[2]. It is endemic in central and southern Europe, with the most common species being *Brucells melitensis*, *Brucella abortus*, and *Brucella suis *[3]. The disease is related to a broad spectrum of clinical manifestations, affecting multiple organs and systems, like the brain, breast, gastrointestinal, and genitourinary systems [4]. The musculoskeletal system can involve multiple regions, most commonly the spine and the sacroiliac joints, while only a few reports of isolated hip infection have been published in the literature [5, 6].

We present a case of neglected, misdiagnosed chronic brucellosis of the lower spine indirectly affecting the hip joint, mimicking sciatica.

## Case presentation

A 51-year-old Greek male patient was admitted to our clinic, referring to intense headaches and hip pain lasting for 12 hours. His medical history was significant for an episode of incomplete cauda equina syndrome of unknown etiology, with additional corrosion of the sacrum, dura mater disruption, and cerebrospinal fluid leak. This resulted in a productive sinus ending posteriorly to the right greater trochanter nine years ago. After that, he was treated with a lumboperitoneal valve implantation, but two years later, he developed a soft tissue infection at the sinus outfall. The radiological evaluation revealed multiple abscesses of the lower lumbar spine and the S1-S2 vertebrae. The physicians performed a superficial surgical debridement of the soft tissue around the buttock without bony intervention. Despite being an ineffective treatment option, the reason for not proceeding with bone lesion debridement remains unclear. In those cultures, *Staphylococcus aureus* was isolated. Interestingly, at that time, specific cultures for Salmonella spp were negative. We suppose that the doctors tried to connect the infection recurrence with specific bacteria causing sacroiliitis, like Salmonella spp. However, no tests for brucellosis were found in the patient’s medical report at that period. Oral medication with moxifloxacin did not manage to control the infection in the long term, with the patient having multiple surgical soft tissue debridements in the past. Meanwhile, no episodes of meningitis were noticed. 

**Figure 1:**
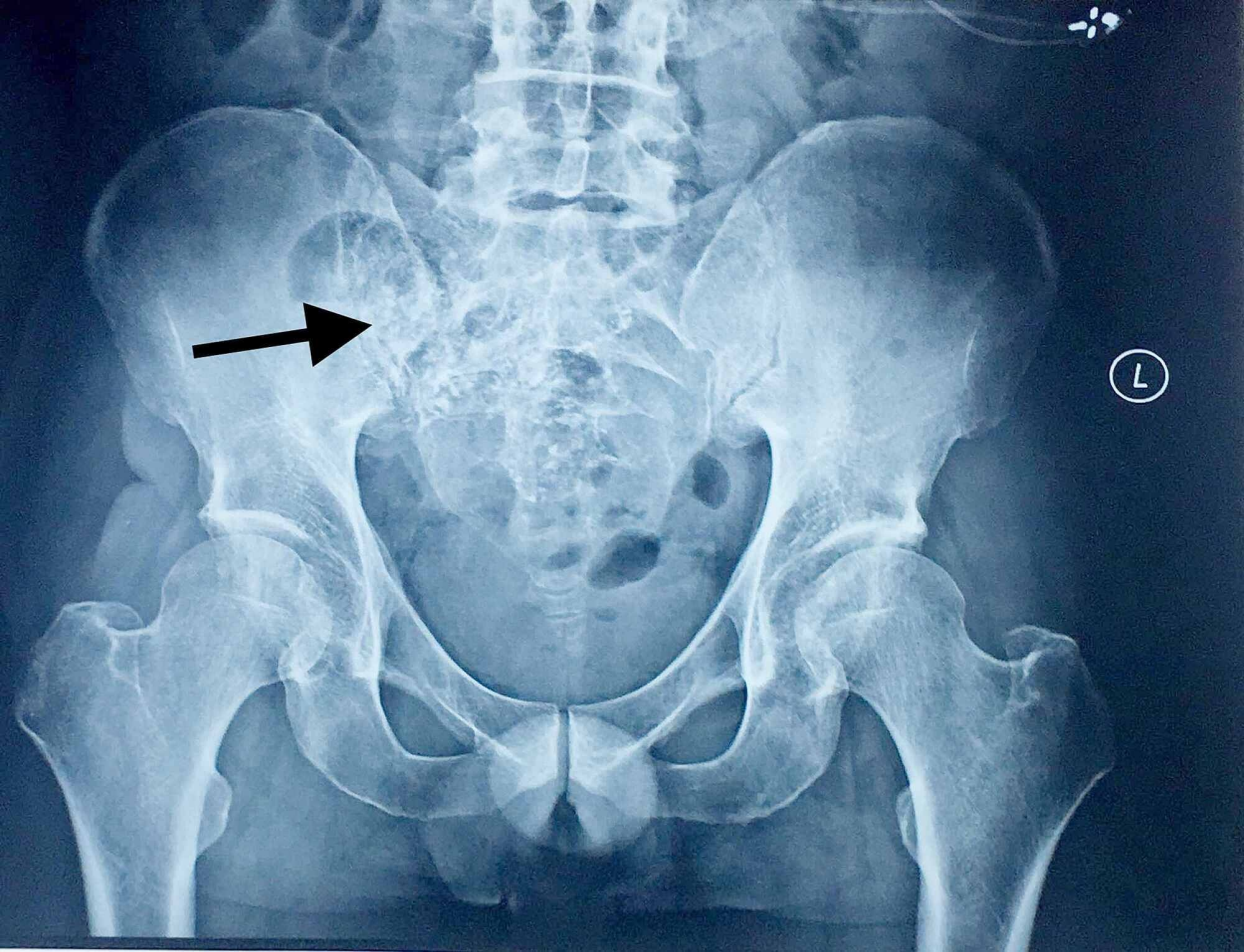
Roentgenography of the pelvis and lower spine. Lesions around the right sacroiliac joint with evident corrosion and an air/fluid collection area indicative of an abscess. An additional soft tissue signaling alteration around the right hip is noticed.

The patient also reported the last hospital visit six months before his current admission with exacerbating hip pain and excessive sinus fluid production. The right hip joint was typical on plain roentgenography, with multiple sclerotic and osteolytic lesions noted on the right sacroiliac joint (Figure 1).

**Figure 2:**
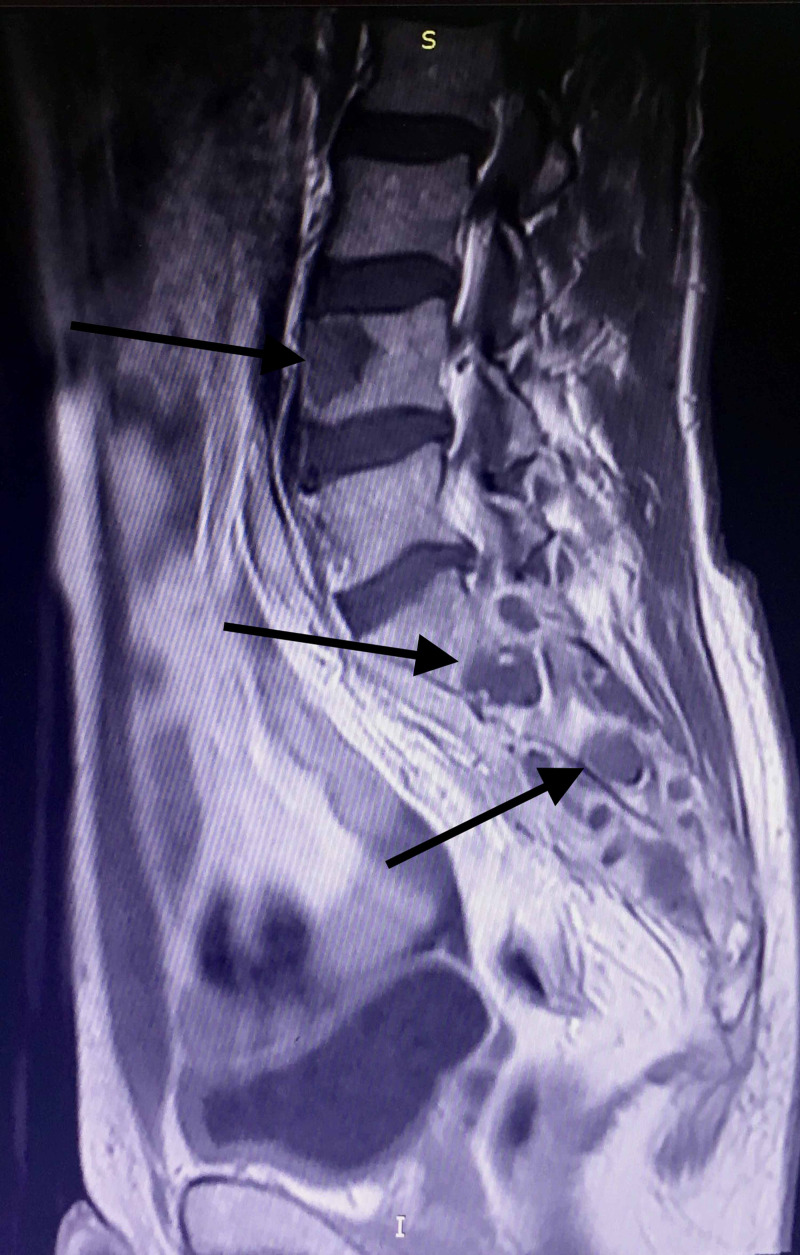
MRI findings, including cystic lesions in the right sacroiliac joint and the L4 vertebra.

The magnetic resonance imaging (MRI) with intravenous contrast agent (gadoteric acid) revealed multiple osteolytic lesions of the right sacroiliac joint and the L4 vertebra (Figure 2).

**Figure 3:**
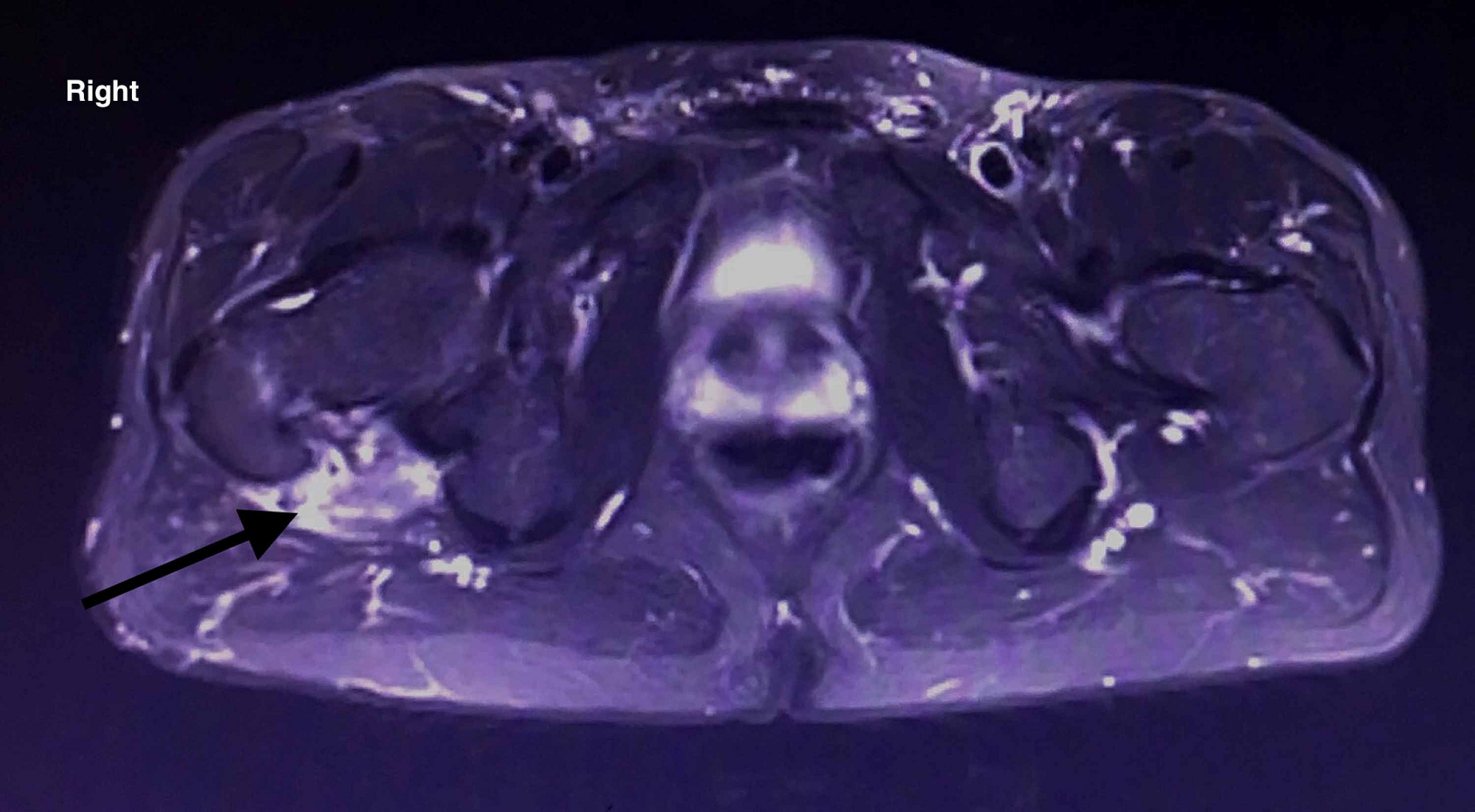
MRI of the lower spine and pelvis. Sinus tract imaging in the transverse plane. The posterior hip capsule is affected

**Figure 4:**
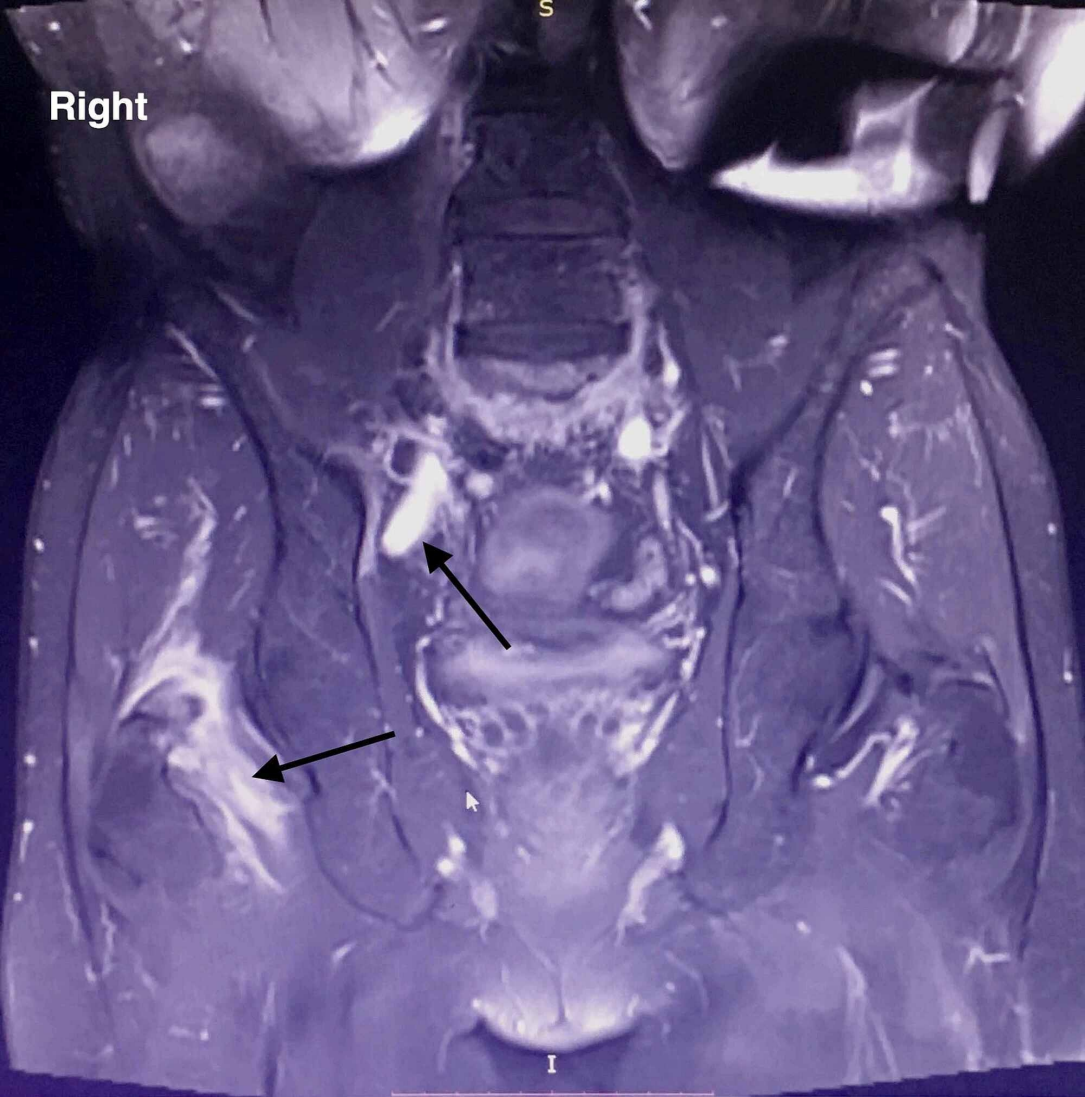
MRI of the lower spine and pelvis. Radiological imaging of sinus tract in the coronal plane showing posterior sinus penetration around the hip joint.

**Figure 5:**
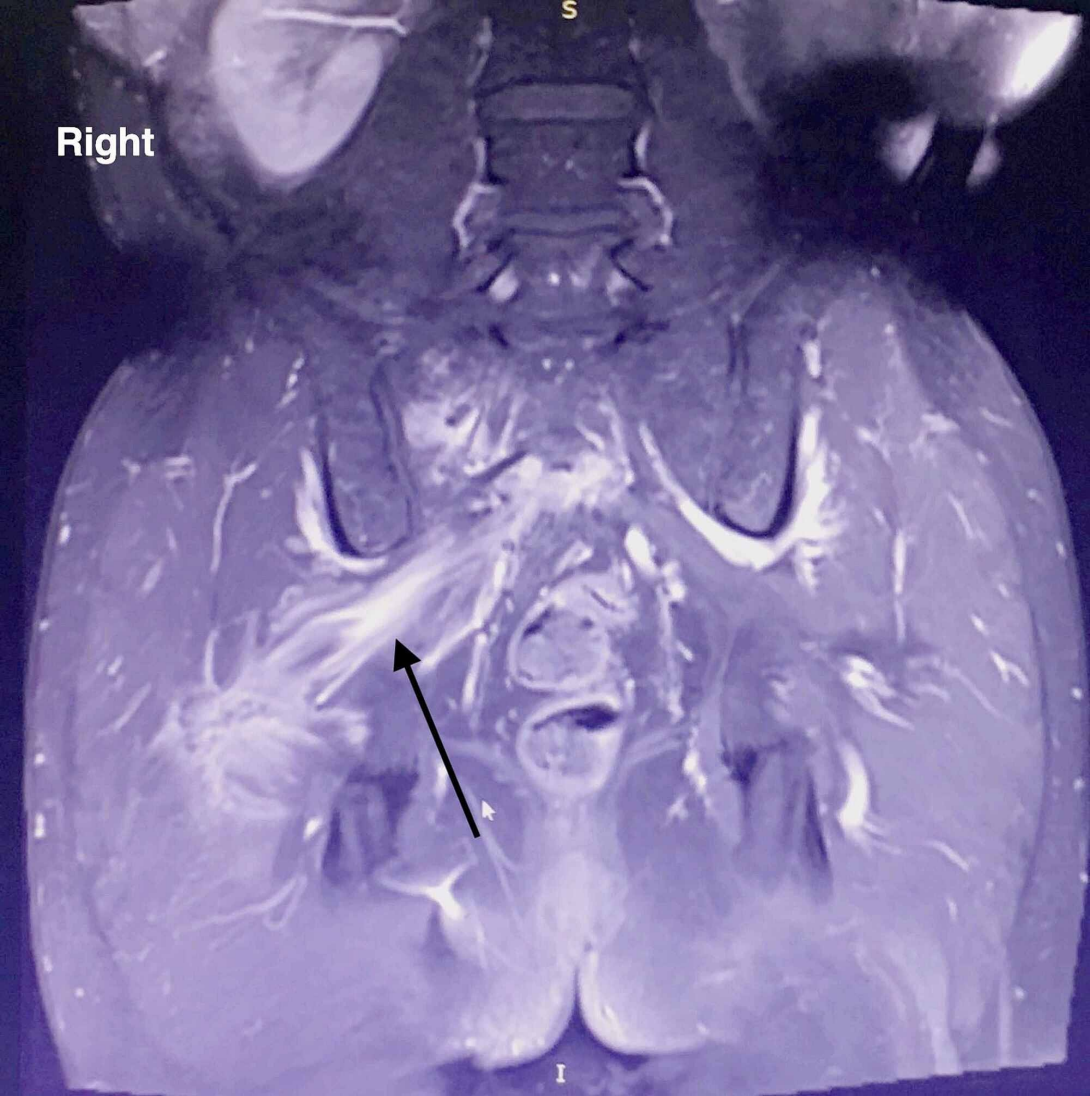
MRI of the lower spine and pelvis. Radiological imaging of sinus tract in the coronal plane showing posterior sinus penetration around the hip joint.

Evaluating the former MRI imaging from the patient's medical file, the epidural sinus was notably expanded around the right hip joint affecting the piriformis muscle belly and tendon, and finally communicating with the joint capsule (Figures 3, 4, 5)

Despite the alteration of the imaging findings, the patient was treated conservatively with blinded intravenous administration of ciprofloxacin and clindamycin and oral celecoxib. This conservative treatment scheme six months ago managed to improve his symptoms until today.

On our clinical examination, a posterior thigh scar and a sinus just posteriorly to the greater trochanter were noted.

Lumbar spine range of motion (ROM), leg muscle strength, reflexes, and sensation of both limbs were normal. The patient had significant gait difficulty with intolerable weight-bearing, a positive Trendelenburg sign on the right hip, and a painful log-roll test. Hip flexion was relatively comfortable at 20°, extension 10°, and abduction/adduction of 20°. At that time, the sacroiliac joint's clinical evaluation was misleading as the FABER test could not be performed, and distraction/compression tests were controversial.

**Figure 6:**
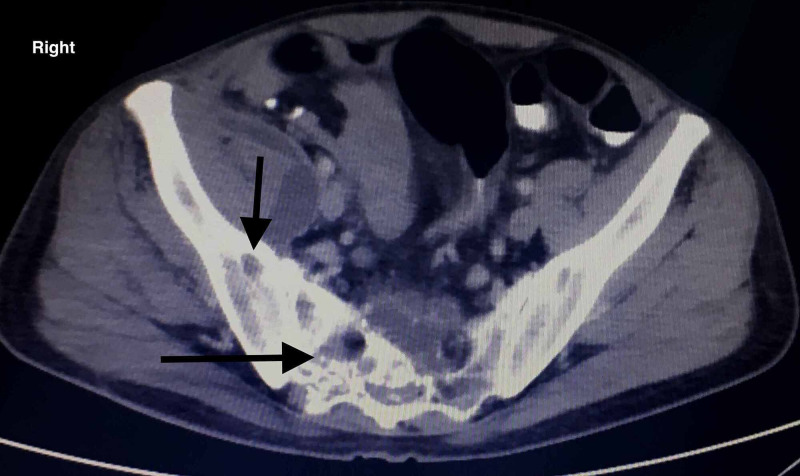
CT evaluation of the pelvis. The sacrum and the right sacroiliac joint are both affected with noticeable corrosions.

**Figure 7:**
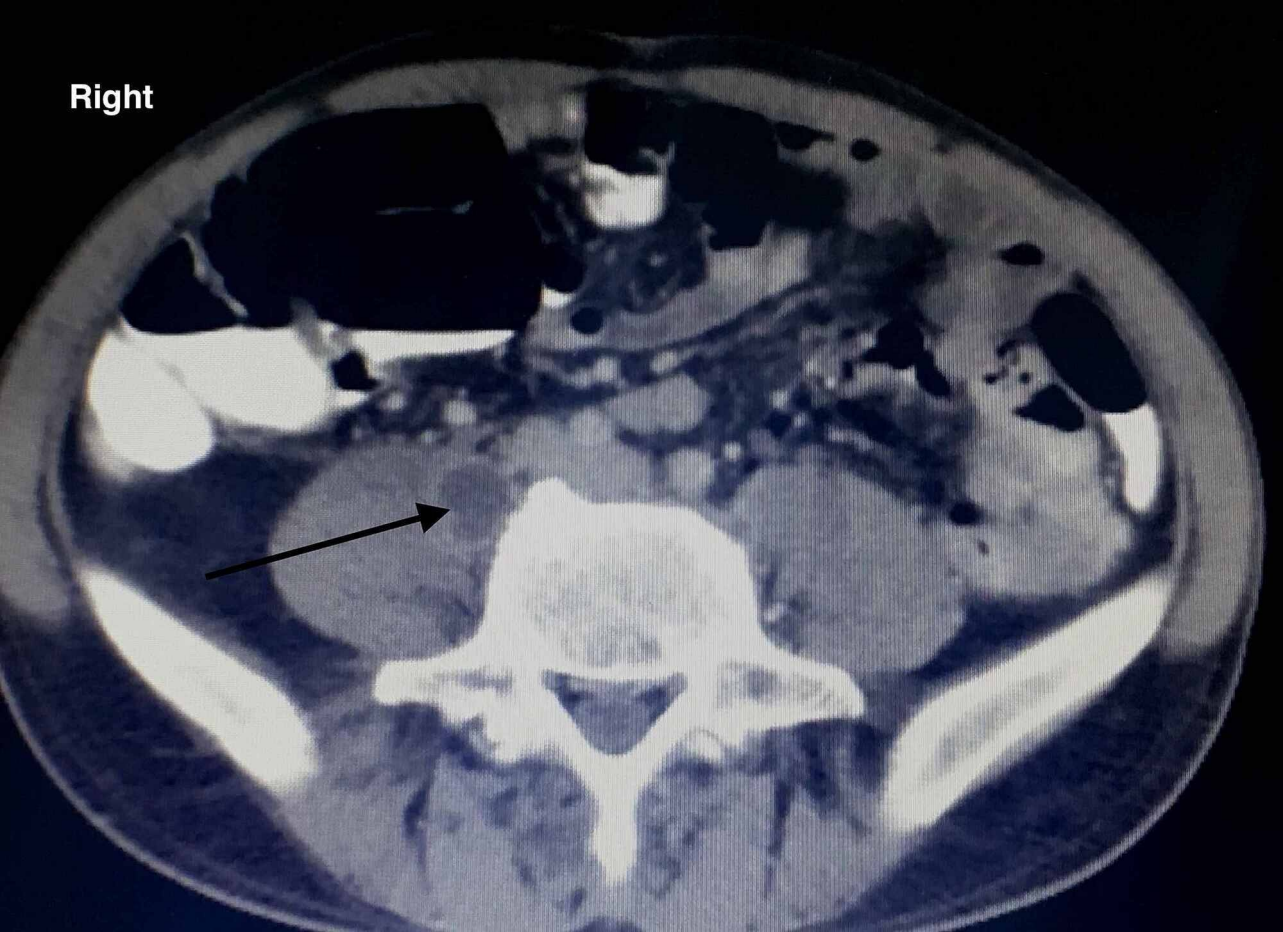
CT evaluation of the pelvis. An abscess of the right iliopsoas muscle is noticed, indicating the local spread of the infection.

On our radiological evaluation, brain computed tomography (CT) was standard, while anterior hip soft tissue ultrasound revealed a newly formed cavity communicating with the regional sinus. The CT of the lumbar spine and hip confirmed the former findings revealing additional intramuscular multiple abscesses of the psoas (Figures 6, 7)

**Figure 8:**
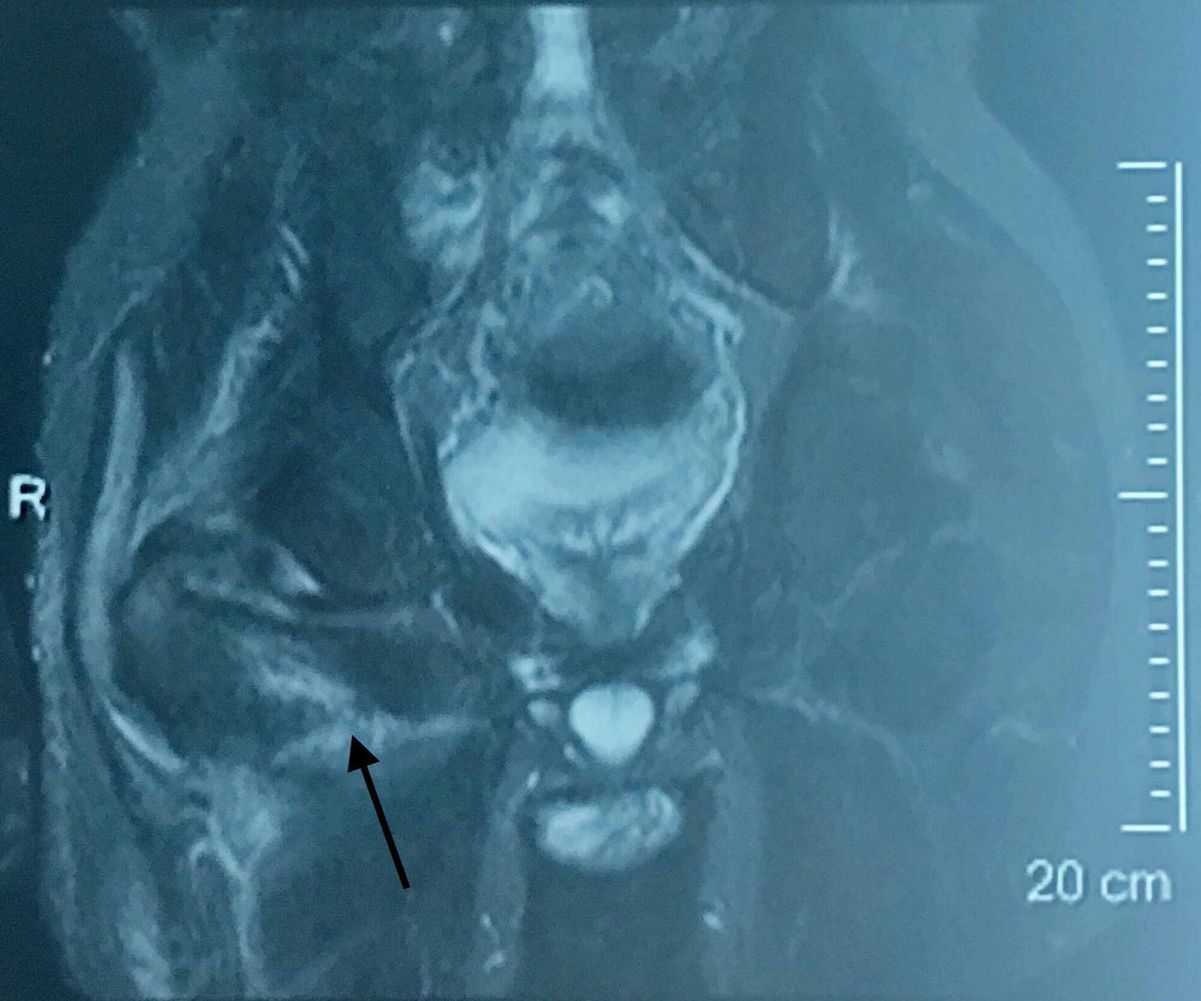
MRI evaluation after medication (coronal plane). Sinus tract signaling is improved around the hip joint while no chondral lesions are noticed.

**Figure 9:**
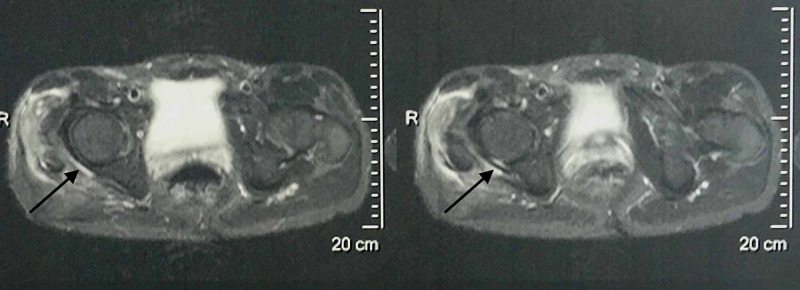
MRI evaluation after medication (transverse plane). The posterior hip capsule signal is improved. The joint space is clear without effusion.

Cerebrospinal fluid (colorless, glucose=60-80% of serum glucose, lactate dehydrogenase (LDH)=10% of serum LDH) and abscess fluid cultures were negative for common bacteria, where no fungal cultures were obtained. Due to radiological imaging characteristics with multiple abscess cavities affecting non-continuous vertebras, in addition to the sacroiliac joint and psoas involvement and culture-negative abscesses, chronic brucellosis was highly suspected. The serum agglutination test (SAT-Wright) was positive, with an SAT titer of 320 (>1/160, prozone phenomenon). We thereafter proposed a surgical debridement and lumboperitoneal valve removal, but the patient denied any further operation. By rejecting every surgical recommendation, we could not obtain different cultures but from the local sinus, which were positive for *Staphylococcus aureus* and *Streptococcus mutans* and negative for Brucella species, results that are indicative for sinus contamination. Thus, we proceeded with oral administration of doxycycline (200 mg/day), ciprofloxacin (800 mg/day), and rifampicin (900 mg/day). Additional administration of celecoxib (200 mg/day) was prescribed. The patient was discharged with instructions for tactical follow-up for clinical examination and inflammatory markers (white blood cells [WBC], erythrocyte sedimentation rate [ESR], C-reactive protein [CRP]) evaluation. After six months of antibiotics administration, his blood laboratory markers remained slightly high with ESR=30 mm/1st h, WBC=12300/uL, and CRP=3,3 mg/dl, but the patient is asymptomatic regarding pain and reports unimportant gait abnormalities. Although the indices remained borderline, the antibiotic scheme reached a maximum duration of six months. Our infectious disease specialist consultation proposed against treatment continuation, under the high possibility of side effects due to the prolonged treatment period. In addition, the patient was informed that in case of a major relapse, radical debridement would be unavoidable. Eventually, subsequent MRI on six-month evaluation revealed improved sinuses tract imaging (Figures 8, 9).

Nonetheless**, **they remainclinically** **productive with a fluid quantity of less than 10ml/day**. **To date, 14 months after the final admission, the patient has no prominent sinus, while the laboratory examination, including ESR and CRP, is regular. He reports significant pain control and gait improvement, and spine and pelvic roentgenography do not show notable alterations. He is instructed for periodic blood test evaluation and is strongly advised to proceed to radical surgical debridement in future disease relapse.

The authors have obtained informed consent about data publication, including photographs, clinical history, and radiological images.

## Discussion

Brucellosis has a regional distribution with a high prevalence in the Balkan peninsula [7]. Acute infection is characterized by systematic symptoms like fever, hepatomegaly, splenomegaly, and arthralgia, but the musculoskeletal system is also involved mainly with spondylitis (3.1%) and sacroiliitis (6.2%) [8]. Adjusting those rates in a cohort of patients with brucellosis and musculoskeletal involvement, Geyik et al. [9] found that sacroiliitis is up to 55% and spondylitis to 31%. More specifically, and as also happened in our case, the L5-S1 segment is commonly affected (31.3%), while epidural extension (56.4%) and cord compression (10.2%) are not uncommon in another study [10]. When peripheral joints are segmentally affected (monoarthritis rates up to 56.1%), the hip (38.6%) and knee (31.9%) joints are the most common locations [11]. Chondral lesions with arthritic changes are reported to rates as high as 41% when the hip is primarily affected [12]. In our case, despite hip involvement, we did not have any evidence of hip arthritis. Hip chondral sparing is probably explained because of an indirect affection of the hip joint by spinal sinuses, which to our knowledge, is an infrequent entity. Our patient reported such sinuses nine years ago after presenting with incomplete cauda equine syndrome and disruption of the sacrum of unknown (up to that time) etiology. This diagnosis and the dura mater disruption were set by radiological evaluation with subarachnoid contrast agent effusion eight years ago, but the results were not in the patient's medical file and thus regarded as controversial. 

Our treatment proposal combined radical surgical debridement and prolonged antibiotics administration, as proposed by the Ioannina recommendations [13]. To our long experience in bone infection, surgical debridement is the cornerstone treatment, while debridement with interbody fusion provides excellent results in spinal brucellosis, according to Abulizi et al. [14]. Nevertheless, despite the comprehensive patient information about this approach's importance, he denied any surgery requiring only medication. 

Many antibiotic combinations have been proposed for brucellosis treatment, with controversial results. In general, monotherapy is no longer indicated due to high relapse rates [9]. Doxycycline (D) is the primary treatment agent, and many combinations have been studied. Rifampicin (R) addition is proposed in uncomplicated brucellosis (DR) with the adequate disease control [15] regarded as the gold standard combination therapy alternatively to streptomycin/doxycycline (DS) combination [16]. Respecting the role of streptomycin, though, despite DS offering better disease relapse control [17], it is less tolerant than rifampicin and indicated in cases expected to require short-term medication. In order to eliminate relapse rates, combinations of three well-tolerated medical agents have been proposed. Ciprofloxacin addition to the DR combination seems to give optimal results showing non-inferiority against DS alone [18]; even though agents like hydroxychloroquine have also been studied as additional to DR combination [19], we preferred ciprofloxacin (DRC) due to our limited experience on hydroxychloroquine adverse reactions and final relapse control. Finally, after choosing the DRC treatment, we also administered celecoxib. The non-steroidal anti-inflammatory drugs (NSAIDs) had two options in our case. They act as painkillers and potent anti-inflammatory agents, limiting the affected hip symptoms and improving gait abnormalities. Secondly, chronic brucellosis (as every chronic inflammatory condition) is characterized by elevated cytokines like interleukin (IL)-1, IL-6, tumor necrosis factor (TNF)-α, and CRP. Those cytokines are also elevated in patients with chronic depression, and celecoxib is found to be a fair anti-depressant treatment in patients with brucellosis acting directly on that path [20]. 

## Conclusions

In conclusion, chronic brucellosis is a disease with high rates in the Balkan region. Spinal involvement is widespread, mainly affecting the L4 vertebra and the sacroiliac joints. Isolated extra spinal manifestations are rare, but physicians should be highly suspicious when medical history is positive of brucellosis cases in the patient's family. To our knowledge, indirect hip infection through advancing sinuses is not reported in the literature. Surgical treatment is not mandatory, but it is highly advised in cases of conservative treatment failure, and standard medicine combinations should be preferred over monotherapy.

## References

[1] Pappas G, Akritidis N, Bosilkovski M, Tsianos E (2005). Brucellosis. N Engl J Med.

[2] Al-Eissa YA (1999). Brucellosis in Saudi Arabia: Past, present and future. Ann Saudi Med.

[3] Taleski V, Zerva L, Kantardjiev T (2002). An overview of the epidemiology and epizootology of brucellosis in selected countries of Central and Southeast Europe. Vet Microbiol.

[4] Al-Nakshabandi NA (2012). The spectrum of imaging findings of brucellosis: a pictorial essay. Can Assoc Radiol J.

[5] Ortega-Andreu M, Rodriguez-Merchan EC, Aguera-Gavalda M (2002). Brucellosis as a cause of septic loosening of total hip arthroplasty. J Arthroplasty.

[6] Salarvand S, Nazer M, Shokri S, Bazhvan S, Pournia Y (2012). Brucellosis-induced avascular necrosis of the hip in a middle-aged person. Iran J Public Health.

[7] Pappas G, Papadimitriou P, Akritidis N, Christou L, Tsianos EV (2006). The new global map of human brucellosis. Lancet Infect Dis.

[8] Zheng R, Xie S, Lu X, Sun L, Zhou Y, Zhang Y, Wang K (2018). A systematic review and meta-analysis of epidemiology and clinical manifestations of human brucellosis in China. Biomed Res Int.

[9] Geyik MF, Gur A, Nas K, Cevik R, Sarac J, Dikici B, Ayaz C (2002). Musculoskeletal involvement of brucellosis in different age groups: a study of 195 cases. Swiss Med Wkly.

[10] Bozgeyik Z, Aglamis S, Bozdag PG, Denk A (2014). Magnetic resonance imaging findings of musculoskeletal brucellosis. Clin Imaging.

[11] Bosilkovski M, Krteva L, Caparoska S, Dimzova M (2004). Osteoarticular involvement in brucellosis: study of 196 cases in the Republic of Macedonia. Croat Med J.

[12] Zaks N, Sukenik S, Alkan M, Flusser D, Neumann L, Buskila D (1995). Musculoskeletal manifestations of brucellosis: a study of 90 cases in Israel. Semin Arthritis Rheum.

[13] Ariza J, Bosilkovski M, Cascio A (2007). Perspectives for the treatment of brucellosis in the 21st century: the Ioannina recommendations. PLoS Med.

[14] Abulizi Y, Liang WD, Muheremu A, Maimaiti M, Sheng WB (2017). Single-stage transforaminal decompression, debridement, interbody fusion, and posterior instrumentation for lumbosacral brucellosis. BMC Surg.

[15] Alavi SM, Alavi L (2013). Treatment of brucellosis: a systematic review of studies in recent twenty years. Caspian J Intern Med.

[16] Solera J, Rodriguez-Zapata M, Geijo P (1995). Doxycycline-rifampin versus doxycycline-streptomycin in treatment of human brucellosis due to Brucella melitensis. The GECMEI Group. Grupo de Estudio de Castilla-la Mancha de Enfermedades Infecciosas. Antimicrob Agents Chemother.

[17] Cisneros JM, Viciana P, Colmenero J, Pachon J, Martinez C, Alarcon A (1990). Multicenter prospective study of treatment of Brucella melitensis brucellosis with doxycycline for 6 weeks plus streptomycin for 2 weeks. Antimicrob Agents Chemother.

[18] Ulu-Kilic A, Karakas A, Erdem H (2014). Update on treatment options for spinal brucellosis. Clin Microbiol Infect.

[19] Majzoobi MM, Hashemi SH, Mamani M, Keramat F, Poorolajal J, Ghasemi Basir HR (2018). Effect of hydroxychloroquine on treatment and recurrence of acute brucellosis: a single-blind, randomized clinical trial. Int J Antimicrob Agents.

[20] Jafari S, Ashrafizadeh SG, Zeinoddini A, Rasoulinejad M, Entezari P, Seddighi S, Akhondzadeh S (2015). Celecoxib for the treatment of mild-to-moderate depression due to acute brucellosis: a double-blind, placebo-controlled, randomized trial. J Clin Pharm Ther.

